# Absence of Asymptomatic Malaria Infection in a Cross-sectional Study in Iranshahr District, Iran under Elimination Programmes

**Published:** 2017

**Authors:** Sakineh PIRAHMADI, Sedigheh ZAKERI, Ahmad RAEISI

**Affiliations:** 1. Malaria and Vector Research Group (MVRG), Biotechnology Research Center (BRC), Pasteur Institute of Iran, Tehran, Iran; 2. National Programme Manager for Malaria Control, Ministry of Health and Medical Education, Tehran, Iran

**Keywords:** *Plasmodium*, Asymptomatic malaria, Elimination, Iran

## Abstract

**Background:**

Asymptomatic malaria infection provides a reservoir of parasites, causing the persistence of malaria transmission. It accounts an important challenge for successful management of the control, elimination, and eradication programmes in any malaria-endemic region. This investigation was designed to assess the presence and the prevalence of asymptomatic carriers in Iranshahr district of Sistan and Baluchistan Province (2013–2014), with a considerable population movement, during the malaria elimination phase in Iran.

**Methods:**

Finger-prick blood samples were collected from symptomless (n=250) and febrile (n=50) individuals residing in Iranshahr district, easthern Iran (Hoodian, Mand, Chah-e Giji, Jolgehashem, Esfand, Dalgan and Chahshour) during Jan 2013 to Dec 2014, and *Plasmodium* infections were detected using light microscopic and highly sensitive nested-PCR techniques.

**Results:**

Thick and thin Giemsa-stained blood smears were negative for *Plasmodium* parasites. In addition, based on nested-PCR analysis, no *P. vivax*, *P. falciparum,* and *P. malariae* parasites were detected among the studied individuals.

**Conclusion:**

Investigation the absence of asymptomatic carriers in Iranshahr district was illustrated and achieving malaria elimination in this area is feasible in a near future.

## Introduction

Malaria elimination is a significant international goal, and many endemic countries are making their attempt to be classified as a malaria-free country to obtain WHO certificate. Based on WHO report, 19 countries are in pre-elimination or elimination and seven countries in the prevention of malaria reintroduction phases (1). Currently, the scale-up of interventions has reduced malaria burden and transmission in many malaria-endemic countries (2–4); hence, transmission often becomes focal (5), and control and elimination programmes need to detect and target the parasite reservoirs. Therefore, to achieve successful elimination programmes in given malaria-endemic areas, important challenges such as the importation of parasite through population movement, the presence of asymptomatic cases, the spread of insecticide, and antimalarial drug resistance urgently require to be addressed (6–9).

Asymptomatic *malaria* infection is defined as the existence of *Plasmodium* parasites in peripheral blood in the absence of symptoms in a given population. It accounts one of the main challenges for the management of the elimination programmes due to providing a reservoir for continuous transmission of parasite to the anopheline mosquitoes and subsequently causing persistence of malaria transmission (10–13). Therefore, to attain a successful elimination, early detection and treatment of all parasite carriers, including symptomatic and asymptomatic cases, is essential. Moreover, all malaria control programmes have passive surveillance systems to identify and treat the infected individuals with malaria who come and seek for medical care in health facilities. However, this method of detection has alone a limited impact on malaria control as only symptomatic but not asymptomatic patients receive treatment. These asymptomatic carriers, including those who are carrying gametocytes (the life parasite stage responsible for transmission to mosquitoes), can act as a source of parasite reservoirs among a population (10, 14–16). Consequently, to defeat this challenge and to target the asymptomatic carriers, as well as symptomatic infections in individuals who do not or cannot seek treatment, active case detection (ACD) strategies were recommended (17). However, the selection of ACD type should be based on the transmission setting as well as geographic and demographic risks (18).

Moreover, asymptomatic malaria infection is not often detectable by routine laboratory techniques. Although light microscopy is the gold standard and can detect parasite species and densities in any control and elimination settings (19, 20), the existence of asymptomatic and low parasitaemia infections is often beyond the detective capacity of microscopic diagnosis (21–23). Furthermore, the detection of sub-patent infections requires sensitive molecular diagnostic methods, such as polymerase chain reaction (PCR) or loop-mediated isothermal amplification (LAMP) (24–27). However, in all malaria-endemic settings, applying both of these techniques for ACD is impractical because of their cost and infrastructure requirements. In addition, the use of these methods will certainly increase the proportion of detected sexual and asexual sub-microscopic infection carriers treated (28, 29).

In Iran, a country located in south-west of Asia, malaria was a major health problem in the past with approximately 96340 cases in 1991, which gradually dropped to 15712 in 2007. Therefore, Iran started its malaria elimination programs in 2009 (30), and more reduction was observed in malaria cases during pre-elimination and elimination phases from 3031 in 2010 to 1373 total cases in 2013 (1). This reduction in malaria cases was due to the scaling-up of different interventions (1) by Iranian national malaria strategic plan through indoor residual spraying (IRS), distribution of long-lasting impregnated bed nets (LLINs), ACD, case management with artemisinin combination therapy (ACT) combined with improved diagnostic capacities in health facilities, and all were employed with a greater rate in comparison with the malaria control programme. Through these efforts, local *P. falciparum* transmission would be eliminated by 2015, and local transmission of *P. vivax* will be reduced to less than 895 cases by 2016, and finally, malaria elimination from the whole country will be achieved by 2025 (31, 32).

Indeed, to achieve this success, one of the key challenges could be missing infections that could act as a parasite reservoir among a population. Therefore, this investigation, which is the continuation of our previous works (33, 34), was carried out in Iranshahr district in Sistan and Baluchistan province (with the highest reported malaria cases in Iran) for assessment of the presence and the prevalence of asymptomatic carriers. Thus, in light of malaria elimination in Iran, we applied sensitive detection tools such as nested-PCR, to understand better local malaria epidemiology. Knowledge about the prevalence of asymptomatic malaria parasite would facilitate malaria elimination programmes in Iran as well as in the region.

## Material and Methods

### Study areas, sample size calculation and blood sample collection

**Fig. 1:**
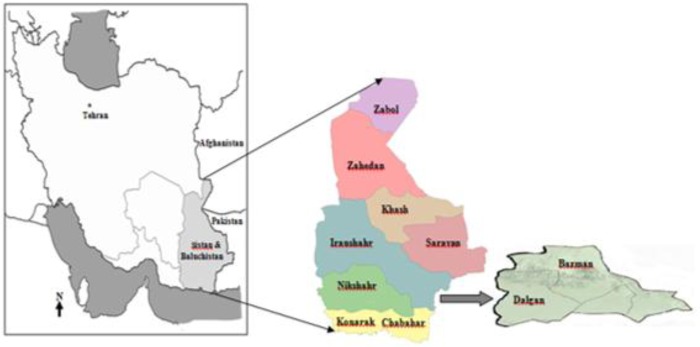
The map of Iran showing the study areas (Hoodian, Mand, Chah-eGiji, JolgeHashem, Esfand and DalganVillages are located in Dalgan County and Chahshour Village is located in Bazman County)

The current study was carried out in Iran-shahr district in Sistan and Baluchistan, a province with the highest reported malaria cases in Iran. This province is located in the southeastern part of Iran with the largest borderline with Afghanistan and Pakistan (Fig. 1). Precipitation is very low and mostly falls in winter. In Iranshahr, malaria transmission is a year-round with two peaks during March to May and September to November with *P. vivax* as the predominant (98.5%) and *P. falciparum* as the second prevalent species (35). The most important anopheles species in this area are *Anopheles culicifacies* and *An.stephensi* (35).

The sample size was calculated using openEpi (36) with 95% confidence level. The study (35) on the prevalence of asymptomatic cases in Iranshahr detected only 0.7% asymptomatic cases in Afghani immigrants but not Iranian. However, if assume that 3% is the minimum prevalence of asymptomatic cases in any malaria-endemic area, the required sample size would be 45. In order to consider the missing data, the sample size was increased to 300. Therefore, in the present cross-sectional survey, 300 individuals were enrolled from different parts of the Iranshahr district in Sistan and Baluchestan province (Fig. 1) from Jan 2013 to Dec 2014. This area was considered because of its endemic situation for malaria also its borderline with Pakistan with a considerable population movement (35, 37). Therefore, this district is vulnerable to receive imported malaria infection from malaria-endemic areas from neighboring countries as well as within the province that may not seek treatment and act as a reservoir of infection in the areas.

The exclusion criteria in this investigation were non-consenting individuals, patients received anti-malarial therapy in the past four weeks prior to the sampling, pregnant women as well as children under two years old did. A written informed consent was obtained from all patients (adults or parents or legal guardians of children), and an ethics approval was obtained from the Ethical Review Committee of Research in Pasteur Institute of Iran. After we obtained an informed consent form, thick and thin blood smears were prepared from all of the participants by a well-trained microscopist in the local malaria health centers.

For detection of sub-patent *Plasmodium* species by nested-PCR (38), a pre-treatment finger-prick blood samples blotted on Whatman 3-mm filter papers (Whatman International, Ltd., Maidstone, England) were dried and stored in the dark at room temperature in small, separate, sealed plastic bags. After collection, the coded filter paper samples were transported to Malaria and Vector Research Group (MVRG) at Pasteur Institute of Iran for molecular diagnosis.

### Detection of Plasmodium species by microscopic and molecular methods

For microscopic examination, thick and thin blood smears were prepared from finger-prick samples. All the slides were air-dried, fixed using methanol in the case of thin blood film and then stained with 1:10 dilution of Giemsa (pH=7.2) for 15–30 min. The stain was washed off with tap water, and the slides were read by local expert microscopists using routine methods;×100 oil immersion lens at ×1000 magnification, at the local health centers in the study areas. The initial slides were considered negative if no parasites were observed after examining 200 fields of Giemsa-stained thick blood smears or counting 1000 red blood cells (RBCs) in a Giemsa stained thin blood film using ×100 oil immersion lens at ×1000 magnification under immersion oil.

For molecular assay, parasite genomic DNA of *Plasmodium* parasites was extracted from dried blood 3 mm spot from different part of the filter paper by using Tris-EDTA buffer-based extraction as described previously (39). The DNA extract was kept at 4°C for use within a few days or stored at −20°C until use. DNA from *Plasmodium* species was detected using nested-PCR amplification of the small sub-unit ribosomal ribonucleic acid (18ssrRNA) gene with species-specific primers for *P. vivax, P. falciparum,* and *P. malariae* as described before (38). This assay has ability to detect the presence of one to ten parasites/μL of blood (38).

The positive controls for the amplification of the *P. vivax* and *P. falciparum* species were genomic DNA of an Iranian *P. vivax* infected patient confirmed with microscopy as well as molecular methods and DNA from the cultured strain K1 of *P. falciparum*, respectively. The *P. malariae* genomic DNA, kindly provided by G. Snounou (Universite Pierre & Marie Curie, Paris, France), was used as a positive control in the present study. Three negative controls were also included in each set of amplification reactions (one with no DNA, the second was filter paper cut without blood, and the third one was from the genomic DNA of healthy individuals with no history of malaria, living in non-malarias areas of the country). In order to avoid cross-contamination, we used different sets of pipettes and various work areas for template preparation, Master Mix for PCR preparation, and the addition of template to the first and second nests.

Each 25 μL reaction mixture for the first and second amplifications contained 2mM MgCl_2_, 200 μM dNTP mixture (Invitrogen, Carlsbad, CA, USA), 1 unit Taq polymerase (Invitrogen, Carlsbad, CA, USA), and a pair of primers (10 pmol each). In addition, 5μL of the extracted DNA and 2 μL of the first PCR product were used as a template in the first and second nested PCR, respectively. Annealing temperature was 58°C for all reactions, and the cycles were repeated 25 and 30 times for the first and the second nested PCR, respectively. The amplified products were resolved by electrophoresis on 2–2.5% agarose gel and stained with ethidium bromide for visual detection by ultraviolet transillumination.

## Results

### Demographic data

To evaluate the presence of asymptomatic malaria cases in Iranshahr district, 300 consent individuals participated in this cross-sectional survey. Of 300 participants, 88.3% were selected randomly with no malaria symptoms; however, the remaining samples (16.7%) were collected from individuals attending the local malaria health centers in Iranshahr district with only fever symptom. The distribution of the age and sex of the participants is shown in Table 1. As shown, 90/300 (30%) males and 210/300 (70%) females, aged between 2 and 74 yr (median age of 19.9), contributed to this work from different villages of Iranshahr district, Sistan and Baluchistan province of Iran.

**Table 1:** Baseline characteristics of the enrolled cases from Iranshahr district in Sistan and Baluchistan Province, Iran

**Iranshahr Villages**	**Sex**	**Median age (Min–Max)**	**Type of sample collection**	**Symptoms**
Hoodian	Female: 37 Male: 13	17.6 (2–74)	Passive	Fever
Mand	Female: 35 Male: 15	14.1 (2–55)	Active	No symptom
Chah-e Giji	Female: 36 Male: 14	18.9 (2–45)	Active	No symptom
Jolgehashem	Female: 37 Male: 13	22.2 (5–67)	Active	No symptom
Esfand	Female: 35 Male: 15	22.6 (2–70)	Active	No symptom
Dalgan	Female: 18 Male: 7	22.3 (2–64)	Active	No symptom
Chahshour	Female: 12 Male: 13	22.1 (2–54)	Active	No symptom

### Results of microscopic examination and Nested-PCR assay

In all 300 thin and thick blood films from both symptomless and febrile cases, no *Plasmodium* parasites were detected. Moreover, no *Plasmodium* species were found using sensitive nested-PCR assay for amplification of 18ssrRNA of *P. vivax, P. falciparum*, and *P. malariae,* which confirmed the microscopy results.

## Discussion

In malaria low-transmission or elimination settings, strategies for detecting and targeting asymptomatic infection become important to reduce the local parasite reservoir and interrupt transmission. Therefore, to achieve successful elimination programmes, ACD is a crucial factor that facilitates prompt detection of asymptomatic cases, which accounts a big challenge in effectiveness and feasibility of elimination strategies. In Iran, malaria transmission is low, and it is present in south and southeastern parts of the country, including Sistan and Baluchistan, Hormozgan and Kerman provinces (31). Hence, the aim of this study was to assess the presences and prevalence of malaria asymptomatic infection (as healthy carriers of parasites) in Iranshahr district of Sistan and Baluchestan province bordering with Pakistan, which is under malaria elimination programme.

Diagnosis of asymptomatic malaria causes challenges due to low level of parasitaemia and no malarial symptoms; as a result, any elimination and eradication efforts may face with difficulties. These carriers may serve as reservoirs of gametocytes and have a key role in continuous malaria transmission (16). Therefore, treatment of asymptomatic malaria cases has been shown to have a valuable role in reduction of malaria transmission in combination with other interventions (40). In the present investigation, to enhance the accuracy and sensitivity of the diagnosis results, microscopic and nested-PCR techniques were simultaneously applied to detect *Plasmodium* parasite in the examined samples. In this cross-sectional survey by using both detection methods, no asymptomatic infections were detected among all symptomless and febrile individuals, and it seems that febrile cases were infected with other pathogens rather than *Plasmodium* parasite. As a result, no hidden parasite reservoirs were detected in this low and a seasonal malaria setting, indicating that it is feasible to achieve successful elimination efforts in these areas.

Indeed, Iranshahr district has shared a borderline with Pakistan, a place with considerable population movement (35, 37) and a high malaria transmission setting. This country implemented its control programmes in order to reduce malaria cases and deaths (1).Therefore, there is a possibility of introducing asymptomatic malaria cases from Pakistan to border districts of Iran, and thus routine monitoring of asymptomatic cases in these areas through ACD is an essential factor in elimination programmes. In fact, having sufficient knowledge in this regard will enable programmes to find the best strategies to control and eliminate malaria in these areas.

In Iran, 90% decrease were reported in the prevalence of total malaria between years 2000 (12294) and 2014 (1251, 4 yr after elimination programme) (37), indeed, through applying massive malaria control tools in these areas after starting malaria elimination in 2010. In 2007, before the employment of elimination strategies in Iran, total malaria cases were 15712, of which 7742 were from Sistan and Baluchistan province. In addition, 549 of 7742 positive cases were from Iranshahr district (37). According to the updated surveillance report in 2014 by the Iranian Center for Disease Management and Control (CDMC, unpublished), the total cases of malaria in Iran were 1251. Of these cases, 331(*P. vivax*) and 39 (*P. falciparum*) mono-infections, as well as 4 mixed infections confirmed by microscopy, were from Iranshahr districts with 276 indigenous cases (CDMC, unpublished). Therefore, although there were considerable non-indigenous malaria cases in this border district, the absence of asymptomatic carriers and decrease in the number of malaria cases confirmed that the achievement of final stage of malaria elimination programme is easily accessible in this malaria-endemic area in a near future. More interestingly, hidden parasite reservoirs are not a big challenge for the elimination campaign in these areas; however, to achieve the final phase of elimination and to enter the eradication phase (malaria free), ACD of symptomatic and asymptomatic carriers with the purpose of proper treatment should be continuously carried out.

Various similar investigations concerning the presence and the prevalence of asymptomatic malaria cases in different malaria endemic settings of Iran have been carried out in the recent years (33–35). In 2011, the presence of the asymptomatic malaria cases among the native residents and Afghani immigrants in Iranshahr district was assessed using light microscopy (35). In this study, no hidden parasites were detected among native residents in Iranshahr; however, 1.6% of Afghani immigrants were *Plasmodium*-positive with no malaria symptoms (35). The prevalence of asymptomatic malaria cases was examined in two malaria endemic settings of Iran (Hormozgan and Kerman provinces) (33), by performing conventional light microscopy, serological and nested-PCR, techniques and they reported the lack of asymptomatic carriers with the evidence of extremely low seropositive to both *P. falciparum* and *P. vivax* parasites. Moreover, using microscopy, serological and nested-PCR techniques in Bashagard District in Hormozgan province no asymptomatic malaria infection was detected in the studied population (34).Therefore, the finding of the present investigation along with the previous studies (33–35) confirmed that there is no asymptomatic malaria infection in malaria-endemic areas of Iran, including Sistan and Baluchistan, Hormozgan, and Kerman provinces and it appears that achieving malaria elimination in Iran is feasible in a near future.

In high-transmission malaria-endemic areas, natural immunity will be acquired after a certain age, as well as after frequent and long-time exposure to parasites (41–44). Indeed, such a natural immunity could protect against malaria clinical episodes but it does not avoid malaria infection; as a result, lead to asymptomatic cases. Asymptomatic malaria infections have been frequently reported in high- and intermediate transmission areas, including Ghana (45,46), Kenya (10), Senegal (47,48), Gabon (49,50), Nigeria (51–53), Uganda (54), Thailand (55), Burma (56), India (57), and Yemen (58). However, in recent years, such cases have been also reported from malaria low-endemic areas, such as Amazon region of Brazil, Peru (16,49–67), Colombia (68), Solomon Island (69), and Principe (70) that was more frequent among adults. All of these reports were in contrast with our previous reports (33, 34) and the present works, in which no asymptomatic carriers were detected in malaria-endemic areas of Iran as a low and seasonal malaria-endemic area. In fact, the presence of asymptomatic *Plasmodium* infection with subpatent parasitaemia results in the persistence of the parasite reservoirs and therefore increases malaria transmission among human populations, which definitely can interfere with malaria elimination strategies. Therefore, to achieve successful elimination and finally, eradication of malaria from the world, survey on the presence and the prevalence of asymptomatic cases in diverse malaria settings are recommended.

## Conclusion

There is no asymptomatic malaria infection in malaria-endemic areas of Iran, including Sistan and Baluchistan, Hormozgan, and Kerman provinces. There is no frequent and long-time exposure of the studied populations to parasites, no natural immunity exists to protect against malaria clinical episodes, therefore, most of the infections are expected to be symptomatic in this area. Moreover, a substantial decrease in malaria prevalence in various malaria-endemic areas of Iran and the absence of asymptomatic cases supported the fact that the current national objective of eliminating malaria can be obtained in a near future. Nevertheless, to avoid any reintroduction of malaria from other malaria-endemic areas through population movement to these areas and to have successful malaria elimination and eradication strategies based on the local epidemiology, routine surveillance of symptomatic and asymptomatic malaria cases in different malaria-endemic areas of Iran is of high concern.
